# Supplementary material for: Structural vulnerability to narcotics-driven firearm violence: An ethnographic and epidemiological study of Philadelphia’s Puerto Rican inner-city
Source: PLoS One. 2019 Nov 21;14(11):e0225376. doi: 10.1371/journal.pone.0225376 (PMC6872141; doi:10.1371/journal.pone.0225376)

**S1 Figure. Continuous Map of Crime and Poverty in Philadelphia.** A version of figure 2 in the main text that uses a continuous color scheme, instead of categorical**,** to highlight outliers. Maps show census tract level counts of crime-related incidents, based on data from the Philadelphia police department, as well as the percent of individuals living under the poverty line and the majority social group from 2012 to 2016 American Community Survey data.


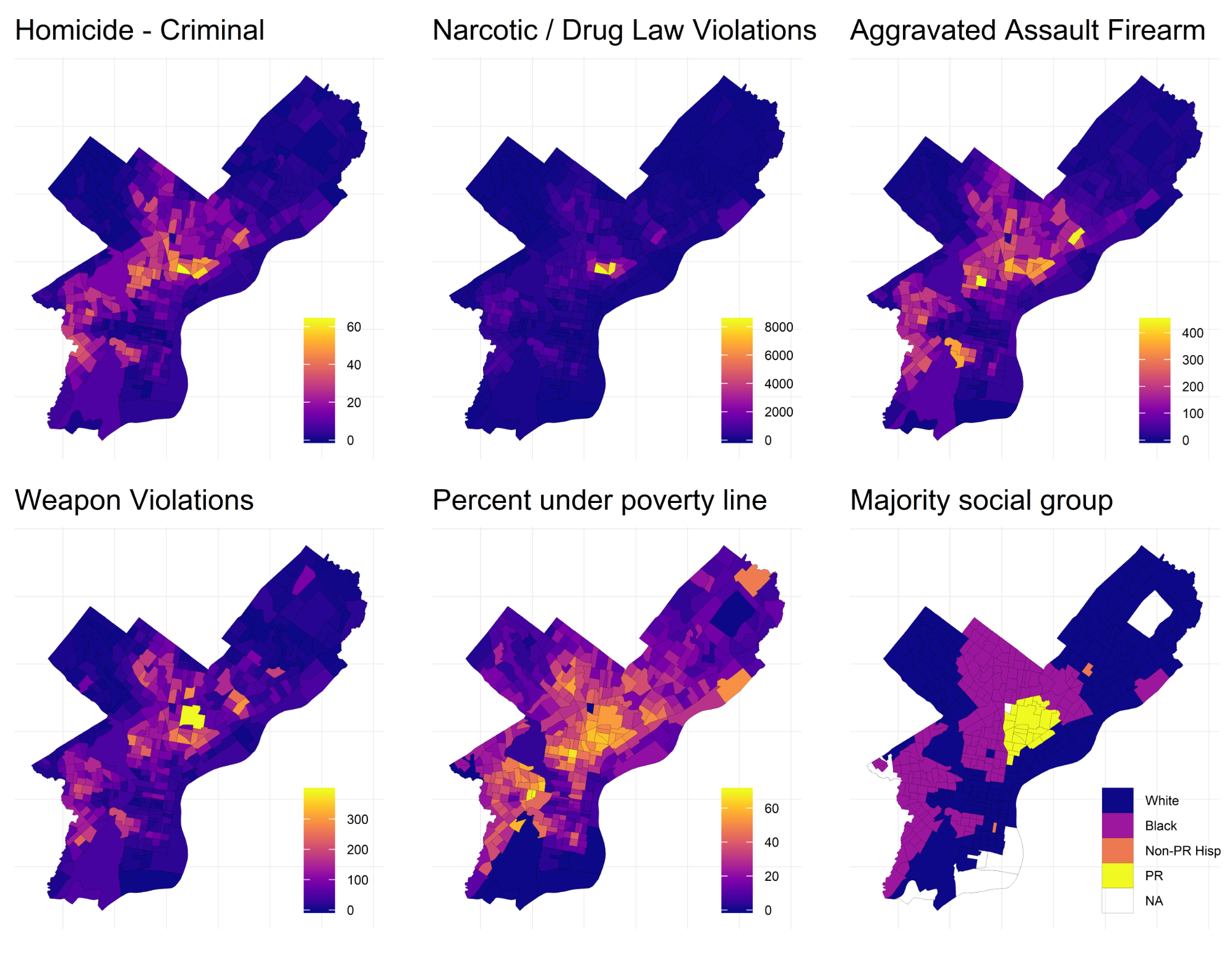

Supplement: S1 Fig — A version of Fig 2 in the main text that uses a continuous color scheme, instead of categorical, to highlight outliers. Maps show census tract level counts of crime-related incidents, based on data from the Philadelphia police department, as well as the percent of individuals living under the poverty line and the majority social group from 2012 to 2016 American Community Survey data. (DOCX) [file pone.0225376.s002.docx]
