# Supplementary material for: Structural vulnerability to narcotics-driven firearm violence: An ethnographic and epidemiological study of Philadelphia’s Puerto Rican inner-city
Source: PLoS One. 2019 Nov 21;14(11):e0225376. doi: 10.1371/journal.pone.0225376 (PMC6872141; doi:10.1371/journal.pone.0225376)
Supplement: Attachment — Submitted filename: R2R_20191008.docx [file pone.0225376.s003.docx]

**Editorial Comments**

In particular, you need to discuss your theoretical framework of structural vulnerability and violence in the first section of the article.

Thank you for your suggestions. We have added the following text to the introduction section, to more thoroughly introduce the structural vulnerability framework on line 103:

*“Our neighbors were rendered vulnerable to violence by structural forces [16–19], such as their low educational levels, marginalization from legal employment opportunities, easy access to wholesale supplies of heroin and cocaine, easy access to licensed and unlicensed firearms, and alienation from social services. Framed in classic public health terminology, each of these circumstances could be considered a social determinant of health—a social factor, out of the individual’s immediate control that modifies their personal risk for adverse health outcomes, such as addiction or death by homicide [20]. Employing the concept of structural vulnerability, however, we can go beyond isolated risk factors to assess how each person’s risk relates to their specific position in the social and economic hierarchies of their local environment [21]. Structural vulnerability is driven by power relations, large-scale social and economic forces that marginalize certain individuals and cause and exacerbate, their illnesses. Beyond just macro-level economic disparities, structural vulnerability also engenders an understanding of how local cultural and social dynamics often mediate or exacerbate personal risk of illness. For example, in our field site we saw how community dynamics and cultural patterns regulate and place limits on the violence occurring in the community. Therefore, any intervention seeking to address violence in inner-city Puerto Rican Philadelphia must be both structurally and culturally competent—it must understand the specific position of community members relative to social factors and structural drivers—if it is to be effective in truly reducing the level of mortality.*

Also reconsider how you label your methodological approach. Is it mixed-methods or multi-methods? Justify your choice by referring to the relevant academic literature.

We understand our research process to be a classic exploratory sequential mixed methods research endeavor, as described by Ozawa and Pongpirul (2014, Health Policy and Planning), Creswell and Plano Clark (2011), and others. In this approach, the first stage of the research is exploratory and qualitative in nature and is used to generate specific hypotheses which can subsequently be tested quantitatively. According to Anguera (2018, Quality and Quantity) this would render our study firmly in the mixed methods category, because it combines both qualitative and quantitative data in the same endeavor. We have added citations in the methods section to support these points as references 25-27.

We have added additional clarifying text detailing the integration of quantitative and qualitative data on line 127:

*“We used an exploratory sequential [25] mixed methods [26,27] study design. In this approach, the first stage entailed an exploratory, ethnographic process by which the ethnographic team deeply acquainted themselves with the field site and the relevant social dynamics. In the second stage, ethnographic data were analyzed using formal qualitative methods. Qualitative conclusions were then used to generate hypotheses about macro-level dynamics that could be tested quantitatively. Epidemiological methods were then applied to quantitative data, in order to assess the hypothesis that had been initially generated ethnographically. Iteratively, qualitative and quantitative results were compared, and placed into dialogue with each other, to produce final conclusions.”*

It is also strange that your ethnographic data yielded qualitative hypotheses about the racial/ethnic and social dynamics of firearm violence in Philadelphia. Rather than hypotheses, did you mean queries/questions that needed further investigation?

We apologize for the confusion, there was a typo in the prior version of the manuscript that may have led to this misunderstanding. We would like to clarify that our research employed qualitative ethnographic methods/data first, which then naturally lead to the creation of *quantitative* (not qualitative) hypotheses. These represented hypotheses in the sense that they required quantitative information and formal statistical testing to establish their relevance to the population that we had previously only studied with ethnography. The word ‘qualitative’ has now been changed to ‘quantitative’ on line 179.

The names Leo and Tito-- are these actual names of research participants or pseudo names used to protect their identities?

These are pseudonyms used to protect participant identities. We have added clarifying text to this effect on line 159:

*“All names describing research participants in this work are pseudonyms.”*

Finally, how many interviews were conducted? How were the interviews analyzed? Where there any differences in views of interviewees in regard to your main research questions?

The precise number of interviews and participants is difficult to ascertain, as the conversational nature of the interviews and participant-observation nature of the work means that interviews often feature numerous individuals, and interviews blend into one another. However, we have provided additional data clarifying this, and describing the database, on line 152:

*“These ethnographic experiences yielded over 1300 pages of field notes, and nearly 2000 pages of transcripts from hundreds of interviews with dozens of respondents, hundreds of photographs, and dozens of hours of video. Our final qualitative database consisted of 3,189 pages of fieldnotes and transcriptions of interviews, representing 471 separate files, which often includes data from multiple participants and overlapping interviews.*

We have added additional text describing the methods for coding and analyzing the interviews, as well as the numerous efforts we undertook to rectify differing views related to our core research questions. We have added the following text in this regard on line 166:

*“All ethnographic data was loaded into the NVivo qualitative analysis platform and coded for themes and characters. Analysis was done iteratively, with preliminary rounds beginning while the fieldwork was still being conducted, which was used to prompt follow-up assessments and refine research questions. Self-reports of behavior were triangulated against qualitative observations of practices occurring in real time in the natural environment. As we began the preliminary analysis, we also further explored the generalizability of our observations in the later stages of our fieldwork. This was accomplished by strategically following up with individuals who had made statements or exhibited behaviors counter to the dominant ideas in the community about research questions. Through long term documentation, we were able to assess whether these "exceptions prove the rule" or if they represented a significant divergence from our findings that required us to shift our analysis. Ultimately, what we present here is a reflection of the dominant social dynamics and views in each neighborhood—those that might have the most impact on the macro-level statistical trends we observe. Final rounds of qualitative analysis occurred until “saturation” had been achieved, at which point the qualitative analysis yielded several quantitative hypotheses about the racial/ethnic and social dynamics of firearm violence in Philadelphia”*

When submitting your revision, we need you to address these additional requirements.

1. Please ensure that your manuscript meets PLOS ONE's style requirements, including those for file naming. The PLOS ONE style templates can be found at

http://www.journals.plos.org/plosone/s/file?id=wjVg/PLOSOne_formatting_sample_main_body.pdf and http://www.journals.plos.org/plosone/s/file?id=ba62/PLOSOne_formatting_sample_title_authors_affiliations.pdf

We have updated the manuscript and figures to meet the style requirements detailed above.

2. Please provide additional details regarding participant consent.

In the ethics statement in the Methods and online submission information, please ensure that you have specified (a) whether consent was informed and (b) what type you obtained (for instance, written or verbal, and if verbal, how it was documented and witnessed).

If your study included minors, state whether you obtained consent from parents or guardians.

If the need for consent was waived by the ethics committee, please include this information.

We have added the following clarifying text to the qualitative methods section to address the questions on line 160:

*“We obtained verbal informed consent because for ethnographic data, the benefits to protecting confidentiality by documenting it with a signature are outweighed by the risk that a hard copy paper signature represents as the most directly-identifying legal marker of the participant. Verbal informed consent was also obtained from parents or guardians for unemancipated minors as well as from the minors themselves.”*

**Reviewer #1:** - This article presents an examination of the relationship between poverty, narcotics, and violence through a mixed-methods, ethnographic and epidemiological assessment, approach in Philadelphia. There are many benefits to this design, which provides rich insight into this issue. The ethnography itself is robust; however, there are some limitations with the quantitative data (discussed below). I cannot comment on the quantitative methods. The article is well-written and with minor revisions could be suitable for publication.
- The theoretical framework is not entirely clear, although it is touched upon in the discussion. The article needs to be situated within this framework early on.

Thank you for your suggestions. We have added text to the introduction section, to introduce the structural vulnerability framework on line 103, as detailed above in our response to the academic editor.

- The article in well written and seem to be free of grammar issues. However, there are some terms or use of jargon, such an “integrated” ethnographic and epidemiological assessment, which are not entirely clear.
- I'm not convinced this is a mixed methods study but a multimethod one. It is not indicated in the methods how authors mixed or used findings to complement each other. As indicated in the abstract, how were methods "integrated" exactly? Or, was it a sequential, multi-method approach? Authors would benefit from citing and explaining the use of these two methodologies more clearly.

For clarity, we have replaced the term “integrated” with “mixed methods” in the abstract, on line 22, which we believe was the only mention in the text. The following text is copied from our response to the academic editor above:

We understand our research process to be a classic exploratory sequential mixed methods research endeavor, as described by Ozawa and Pongpirul (2014, Health Policy and Planning), Creswell and Plano Clark (2011), and others. In this approach, the first stage of the research is exploratory and qualitative in nature and is used to generate specific hypotheses which can subsequently be tested quantitatively. According to Anguera (2018, Quality and Quantity) this would render our study firmly in the mixed methods category, because it combines both qualitative and quantitative data in the same endeavor. We have added citations in the methods section to support these points as references 25-27.

We have added additional clarifying text detailing the integration of quantitative and qualitative data on line 127:

*“We used an exploratory sequential [25] mixed methods [26,27] study design. In this approach, the first stage entailed an exploratory, ethnographic process by which the ethnographic team deeply acquainted themselves with the field site and the relevant social dynamics. In the second stage, ethnographic data were analyzed using formal qualitative methods. Qualitative conclusions were then used to generate hypotheses about macro-level dynamics that could be tested quantitatively. Epidemiological methods were then applied to quantitative data, in order to assess the hypothesis that had been initially generated ethnographically. Iteratively, qualitative and quantitative results were compared, and placed into dialogue with each other, to produce final conclusions.”*

- The results are long and it is easy to lose the point the authors are trying to make. If the editor so wishes, they could request the authors to pare down the findings section. -The integration of the quantitative and qualitative findings seem to be slightly superficial in this mixed methods study. There is more to be said about how your findings using each of these methods support each other.

We have added a number of additions to the methods section to improve the discussion of the integration of qualitative and quantitative methods. In addition to the text on line 127 described above, we also added the following:

On line 180:

*“We tested statistically our main hypothesis that, even controlling for poverty levels, poor majority-Puerto Rican neighborhoods in Philadelphia experience higher levels of violence and narcotics traffic than white or black areas. We also sought to confirm our qualitative understandings of unique properties of the field site, in terms of sociodemographic trends including the degree of marginalization, as well as the uniqueness levels of narcotics and violence-related crime experienced in our community. Quantitative data were sought, where possible, to confirm or refute the understandings we had built ethnographically. “*

On line 228:

*“In the last stage of analysis, the qualitative and quantitative results were synthesized to produce final conclusions. This entailed the comparison of qualitative results and qualitatively-generated hypotheses with quantitative data and results. Whenever possible, qualitative and quantitative data describing similar phenomena were placed in close proximity to one another; e.g. maps describing socioeconomic deprivation placed adjacent to ethnographic descriptions of the same phenomena. Final conclusions were drawn after considering all data, and via numerous rounds of consensus-driven feedback from the ethnographic and quantitative analysis teams.”*

- In the methods, authors indicate a follow up was done in the Spring of 2019. What happened? Why was this done and how did it add value?
- The time gap between data collected and 2019 is not touched on in the discussion about any contextual changes since the data was collected. What do authors think might have changed, or are their findings entirely relevant now?

In hopes of maintaining a longitudinal understanding of the field site and the evolution of social dynamics over time, the ethnographic team has sought to maintain key relationships over time and visit on numerous occasions. Although the intensive fieldwork ended in 2013, we have returned on numerous occasions to perform drop-in ethnographic assessments, most recently in 2019. Each time we return we are interested in noting changes since the completion of the intensive fieldwork. In particular, on the occasion of the spring 2019 follow-up, we were interested in exploring any potential short-term impacts of the criminal justice diversion from incarceration reforms instituted by the new DA Larry Krasner (mentioned in the discussion). Although we note some preliminary evidence of success in this respect, it is far too early to document meaningful change. In sum, our follow-up visits have indicated that the main narratives conveyed in this work are largely still the reality in our field site neighborhood. We have added text in the limitations discussing the temporality of our work on line 725:

“Our work is most representative of the time period during which the intensive fieldwork was conducted (2007 through 2013), although our periodic return visits to the field site indicate that the dynamics described here remain largely predominant.”

- I would like to see the authors engage more with the limitations of the quantitative data collected and could these limitations be used to explain your findings?

We have added additional details to description of the limitations of the police data set on line 708:

*“Our results must therefore be considered in the context of the limitations of these data. It is certain that police data undercount the prevalence of both violence and narcotics, and potentially in irregular ways that could bias the results. Nevertheless, the large magnitude of the disparities presented here make it unlikely that any bias in the data could reverse them, especially given their consistency with our ethnographic observations. Nevertheless, it is possible that a bias in the police force against Puerto Rican or Black neighborhoods could lead to greater police attention in those areas, subsequently overrepresenting violence or drug crime in those areas. Furthermore, the white/non-white disparities observed here may be magnified by racial biases held by police officers, wherein drug trafficking in white areas receives less attention that its equivalent in non-white areas.”*

- The conclusions are particularly effective as they are solutions based.

**Reviewer #2:** This is a terrific, well-written exploration of race, structural violence and drug selling and using.

Good multi-methodologic studies, like this one, are challenging to execute. The collaboration between disciplines and the requisite conferring on style, language, hypothesis, data and word count are challenging from the start. The word count is justifiably high to allow for adequate explanation of both the quant and qual data. It is the price of getting the right balance in the inter-disciplinary collaboration. I wish there were more papers like this one.

A couple of minor points to address:
- There is some repetition in the simple description of the neighborhood as “impoverished” “drug” “lucrative market” etc.

Thank you for brining this to our attention. We have removed a number of such instances.

- Line 575: “Figure 5” might be referring to “6”

We have updated this sentence to refer to “fitted lines” showing “predicted results” of the statistical analysis, in order to indicate more clearly why it makes sense to refer to figure 5 here. This can now be found on line 606:

*“The fitted lines on Fig 5 also show the predicted results of a statistical analysis of the poverty-narcotics gradient, separately by majority social group.”*

- Figure 6 could be explained a bit better.

Thanks for highlighting this gap. We have added the following text to the figure 6 caption:

*“For example, a ratio of 6.1 between whites and Puerto Ricans for narcotics-related crime indicates that majority Puerto Rican areas were predicted to have over 6 times more narcotics crime relative to majority white areas, at a poverty level of 60%.”*

As well as the following text to the results section, on line 607:

*“In Fig 6, we predicted the expected rate of narcotics incidents for a poverty rate of 60%, similar to our field site (Fig 6), and compare the ratios of these predictions for each racial group. This allows us to control for poverty and show the association between race and levels of violence- and narcotics-related crime. The predicted values of narcotics-related crime for highly-impoverished, majority-Puerto Rican neighborhoods were significantly elevated compared to that of majority-black and white areas, with ratios of 4.2 (95% CI: 4.1-4.3) and 6.1 (5.8-6.4) respectively (Fig 6). This indicates that at a poverty level of 60%, majority Puerto Rican areas were predicted to have over 6 times more narcotics crime relative to majority white areas.”*

- The claim made in lines 619-620 does not hold for homicide, with the data presented.

This sentence originally read:

*“Furthermore, in each case majority-Puerto Rican status is associated with significantly elevated levels of violence for highly-impoverished neighborhoods (Figure 6).”*

We have updated this statement to make it more accurate, and more closely reflect what figure 6 actually shows, now found on line 634:

*“ Furthermore, Fig 6 indicates that at a 60% poverty level, majority-Puerto Rican status is associated with significantly elevated levels of violence, for each category of violent crime examined.”*
